# The post-discharge anastomotic leak clock: a risk, trend, and trigger framework for earlier recognition after colorectal surgery

**DOI:** 10.1097/MS9.0000000000005776

**Published:** 2026-10-07

**Authors:** Majid Noaman, Fnu Shoaib, Adnan Khatak, Kaleem Khan

**Affiliations:** a Department of Surgery, Liaquat University of Medical & Health Sciences, Jamshoro, Sindh, Pakistan; b Department of Surgery, Nangarhar University, Jalalabad, Afghanistan

**Keywords:** anastomotic leak, colorectal surgery, failure to rescue, postoperative surveillance, remote patient monitoring

## Abstract

Anastomotic leakage remains a major cause of morbidity, reoperation, and failure to rescue after colorectal surgery. Enhanced recovery pathways have shortened hospitalization, yet clinically important leaks may become apparent after discharge, creating an interval during which subtle deterioration can remain unrecognized. Emerging evidence suggests that delayed recognition, particularly after the onset of sepsis, is associated with worse outcomes. This editorial examines the limitations of relying on operative prevention, isolated clinical features, single inflammatory marker measurements, or unstructured patient-initiated presentation. It proposes a Risk, Trend, and Trigger pathway for closed-loop postoperative surveillance. This framework combines predischarge risk stratification with longitudinal assessment of clinical recovery, vital signs, bowel or stoma function, and C-reactive protein trajectories. Predetermined escalation criteria connect concerning changes with timely surgical review, laboratory assessment, cross-sectional imaging, and source control evaluation. Remote monitoring may extend surveillance into the home, but current evidence primarily supports its feasibility and acceptability rather than its ability to reduce leak-related sepsis or mortality. Technology should therefore complement clinical ownership rather than replace it. Discharge should be treated as an active diagnostic handoff supported by documented clinical baselines, accessible contact routes, explicit response times, and reassessment after negative or indeterminate investigations. Prospective multicenter studies should determine whether this approach reduces diagnostic delay and failure to rescue without increasing unnecessary imaging, alert burden, or inequity.

## Introduction

Colorectal recovery has accelerated; anastomotic leak biology has not. Enhanced recovery pathways and minimally invasive surgery have shortened postoperative hospitalization, but they have not necessarily altered when an anastomotic leak becomes clinically apparent. In a meta-analysis of 25 studies involving 11 144 patients, the median time to diagnosis was 6.9 days, with a reported range of 3–10 days^[^1^]^. Clinically consequential leaks may therefore emerge after discharge, during an interval when early manifestations such as increasing pain, anorexia, fatigue, ileus, and tachycardia may be subtle and easily attributed to expected postoperative recovery. Reliance on fever, generalized peritonitis, organ dysfunction, or septic shock risks identifying leakage only after intra-abdominal infection has become established.

The clinical importance of this surveillance gap is underscored by a 2026 cohort study of 39 175 patients undergoing colon resection^[^2^]^. Among patients with organ-space surgical site infection, which was used as a surrogate for anastomotic leakage, diagnosis after the onset of sepsis was associated with higher rates of reoperation and failure-to-rescue than earlier recognition. Although the retrospective design, the use of a surrogate outcome, and a predominantly male Veterans Affairs population limit causal inference and generalizability, the findings support a clinically important association between diagnostic delay and adverse outcomes. Discharge must therefore become an active diagnostic handoff supported by risk stratification, trajectory monitoring, and predefined escalation, rather than a transfer of diagnostic responsibility to the patient.

### The prevention ceiling

Anastomotic leakage should not be understood as a purely technical failure. Adequate perfusion, minimal tension, secure construction, and viable tissue are essential, but healing also depends on nutritional and immune status, comorbid conditions, local inflammation, and interactions with the intestinal microbiota^[^3^]^. Although microbiome research is biologically compelling, much of the mechanistic evidence remains translational and does not yet support routine microbiome-directed intervention.

Indocyanine green (ICG) fluorescence angiography improves intraoperative visualization of perfusion, but its preventive effect has not been consistent across trials. The Phase 3 AVOID trial found no statistically significant overall reduction in clinically relevant leakage^[^4^]^. Similarly, the 2025 ICG-COLORAL trial, which randomized 1136 patients and excluded low anterior resections, reported leakage in 5.8% of the ICG group and 7.9% of the control group (odds ratio, 0.73; 95% confidence interval, 0.48–1.13)^[^5^]^. A possible benefit was observed in left-sided surgery, but this subgroup finding was not definitive. These results do not negate the selective value of perfusion imaging; rather, they demonstrate that intraoperative assessment cannot be assumed to eliminate postoperative risk. Prevention and rescue are complementary safety systems: the inability to prevent every leak increases, rather than diminishes, the obligation to detect deterioration early.

### The post-discharge blind spot

After discharge, detection depends less on dramatic signs than on recognizing deviation from the expected recovery trajectory. Increasing pain, malaise, anorexia, nausea, ileus, reduced mobility, persistent tachycardia, and altered stoma output are nonspecific and may be normalized by patients as part of convalescence. Analgesics can alter pain perception and bowel function, while antipyretics may attenuate fever. A diverting stoma may reduce the clinical consequences of leakage or the need for reoperation, but it does not eliminate either leakage or the need for continued surveillance^[^6^]^.

This vulnerability is organizational as well as biological. Discharge instructions commonly enumerate warning signs without specifying who will review them, how quickly escalation should occur, or where the patient should present. Routine clinical review may occur after the principal diagnostic window, while responsibility is dispersed among patients, families, primary care clinicians, emergency departments, and surgical teams. Geographic distance, limited health literacy, financial constraints, and restricted digital access may further delay reassessment. Early discharge is not inherently unsafe; unstructured discharge without diagnostic continuity is unsafe.

Remote monitoring may help bridge this interval. Small observational studies using symptom surveys, teleconsultations, and wearable vital-sign monitoring have demonstrated feasibility and patient acceptability, including among rural populations^[^7,8^]^. However, these studies were not designed to establish reductions in leak-related sepsis, reoperation, or mortality. Remote monitoring should therefore be regarded as promising infrastructure rather than a validated solution. Its clinical value will depend on equitable access, actionable thresholds, and clear professional ownership of alerts.

### Detecting trajectory, not a single signal

Early recognition is more likely to arise from convergence across clinical, biochemical, and physiological trajectories than from any single abnormality. Increasing rather than resolving pain, a new analgesic requirement, persistent tachycardia, fever or hypothermia, declining oral intake, vomiting, abdominal distension, delayed bowel recovery, or unexpectedly low or high stoma output should prompt reassessment. Dyspnea, oliguria, weakness, and confusion may indicate systemic deterioration. None of these findings is sufficiently sensitive or specific in isolation.

C-reactive protein (CRP) is most useful as a rule-out and trajectory marker rather than a stand-alone confirmatory test. A 2023 meta-analysis derived approximate CRP thresholds of 159, 114, and 109 mg/L on postoperative days 3, 4, and 5, respectively, with values below these thresholds supporting a reduced probability of leakage^[^1^]^. However, thresholds vary across procedures, populations, assays, and baseline risk. Recent retrospective evidence suggests that a rising or insufficiently declining CRP trajectory may provide greater discrimination than an isolated measurement^[^9^]^. A reassuring CRP value should not override progressive clinical deterioration.

When integrated findings raise suspicion, contrast-enhanced computed tomography (CT) of the abdomen and pelvis is generally the first-line imaging investigation. Intravenous contrast facilitates the evaluation of intra-abdominal collections, bowel perfusion, and other complications, while rectal contrast may improve anastomotic assessment when technically appropriate and safely tolerated^[^10^]^. An early negative or equivocal CT result should not close the diagnostic pathway if the patient continues to deteriorate. Peritonitis, hemodynamic instability, or evolving organ dysfunction should prompt urgent source control assessment rather than repeated investigations that could delay treatment.

Wearable measurements of heart rate, temperature, and activity, combined with patient-reported symptoms, may extend trajectory monitoring into the home^[^7,8^]^. These technologies should support clinical judgment rather than replace direct assessment, professional ownership, or timely human review.

### The risk, trend, and trigger pathway

A practical surveillance model should connect risk recognition with longitudinal assessment and predefined action.

#### Risk

Before discharge, the surgical team should document factors that may justify intensified surveillance, including a low rectal or technically difficult anastomosis, emergency surgery, intra-abdominal contamination, intraoperative perfusion concerns, corticosteroid or immunosuppressive exposure, malnutrition, frailty, substantial comorbidity, major blood loss or transfusion, and an abnormal postoperative clinical or inflammatory course. Social and geographical barriers to urgent reassessment should also inform surveillance intensity. Risk should determine the level of monitoring, not whether monitoring occurs.

#### Trend

Discharge documentation should establish a clinical baseline that includes heart rate, temperature, pain and analgesic requirements, oral intake, abdominal findings, bowel or stoma function, and the most recent CRP value and its direction of change. The expected recovery trajectory should be explained to the patient and, when appropriate, the caregiver. Higher-risk patients could receive structured contact within 24–48 hours after discharge and further reassessment during postoperative days 5–7. This schedule represents a proposed model requiring prospective validation rather than a universal recommendation.

#### Trigger

Within this proposed model, escalation criteria should be defined before abnormal findings occur. Reassuring recovery supports continued surveillance. An isolated mild deviation may warrant same-day clinical review and repeat assessment. Persistent tachycardia, increasing pain, poor oral intake, an abnormal CRP trajectory, or altered bowel or stoma function should prompt surgical assessment, laboratory testing, and consideration of CT. Sepsis, peritonism, organ dysfunction, or rapid deterioration should prompt immediate hospital assessment and source control evaluation.

The pathway becomes closed-loop only when it specifies a responsible surgical team, a direct contact route, explicit response times, documentation of alerts, and confirmation that ordered investigations have been reviewed. Persistent concern after negative or indeterminate imaging must trigger reassessment. An alert without ownership is merely data.

### Implementation and research priorities

Structured surveillance may generate false-positive alerts, unnecessary imaging, additional staffing requirements, and alert fatigue. CRP thresholds remain heterogeneous, and evidence from small remote-monitoring studies may not generalize to patients with limited connectivity, low health literacy, language barriers, or complex postoperative needs. Algorithms should, therefore, function as decision-support tools rather than sources of reassurance or substitutes for clinical review.

A 2025 multicenter study illustrates the potential of integrated prediction models to estimate individualized risk of anastomotic leakage^[^11^]^. Predictive performance alone, however, is clinically insufficient unless the model is linked to an explicit response pathway. Prospective multicenter studies should compare structured surveillance with routine discharge and evaluate the interval from the first abnormal signal to surgical review, sepsis before leak recognition, leak severity at diagnosis, emergency reoperation, failure to rescue, permanent stoma, readmission, resource use, and patient-reported burden. Digital access and model performance across demographic, socioeconomic, and geographical groups should also be assessed prospectively. The relevant question is not simply whether surveillance predicts leakage, but whether it accelerates effective intervention without producing disproportionate burden or inequity.

## Conclusion

The relevant quality metric should not be limited to whether an anastomotic leak occurred, but should include whether the health system recognized and treated it before physiological collapse. Enhanced recovery after surgery has successfully shortened inpatient recovery; postoperative surveillance must now evolve beyond the hospital walls. A risk-stratified, trajectory-based, and trigger-driven pathway offers a practical framework for converting discharge from a diagnostic blind spot into a period of active surgical continuity. Prospective studies should determine not only whether structured remote monitoring predicts leakage, but also whether it shortens the interval to surgical review and source control, limits progression to sepsis, and reduces failure to rescue.

## Ethical approval

Not applicable.

## Consent

Not applicable.

## Data Availability

No new data were generated or analyzed for this editorial. All information discussed is derived from previously published literature cited in the manuscript. Data sharing is therefore not applicable.

## Research registration unique identifying number (UIN)

Not applicable. This article is an editorial and does not report a prospective research study, clinical trial, or systematic review requiring registration.

## References

[1] Bona D Danelli P Sozzi A. C-reactive protein and procalcitonin levels to predict anastomotic leak after colorectal surgery: systematic review and meta-analysis. J Gastrointest Surg 2023;27:166–79. 10.1007/s11605-022-05473-z 36175720

[2] Savitch SL Lagisetty KH Suwanabol PA. Delayed diagnosis of anastomotic leak and failure to rescue after colon resection. JAMA Surg 2026;161:352–59. 10.1001/jamasurg.2025.6551 PMC12895316 41671012

[3] Hajjar R Gonzalez E Fragoso G. Gut microbiota influence anastomotic healing in colorectal cancer surgery through modulation of mucosal proinflammatory cytokines. Gut 2023;72:1143–54. 10.1136/gutjnl-2022-328389 36585238

[4] Faber RA Meijer RPJ Droogh DHM. Indocyanine green near-infrared fluorescence bowel perfusion assessment to prevent anastomotic leakage in minimally invasive colorectal surgery (AVOID): a multicentre, randomised, controlled, phase 3 trial. Lancet Gastroenterol Hepatol 2024;9:924–34. 10.1016/S2468-1253(24)00198-5 39151436

[5] Rinne JKA Huhta H Pinta T. Indocyanine green fluorescence imaging in prevention of colorectal anastomotic leakage: a randomized clinical trial. JAMA Surg 2025;160:486–93. 10.1001/jamasurg.2025.0006 PMC11883591 40042831

[6] Myrseth E Nymo LS Gjessing PF. Diverting stomas reduce reoperation rates for anastomotic leak but not overall reoperation rates within 30 days after anterior rectal resection: a national cohort study. Int J Colorectal Dis 2022;37:1681–88. 10.1007/s00384-022-04205-8 PMC9262798 35739403

[7] Dornbush C Mishra A Hrabe J. Remote monitoring after elective colorectal surgery, a pilot study. Surgery 2025;179:108791. 10.1016/j.surg.2024.08.025 39307673

[8] Leenen JPL Ardesch V Patijn G. Remote home monitoring of continuous vital sign measurements by wearables in patients discharged after colorectal surgery: observational feasibility study. JMIR Perioper Med 2023;6:e45113. 10.2196/45113 PMC10199380 37145849

[9] Niemann BR Murthy J Breinholt C. Postoperative C-reactive protein trend is a more accurate predictor of anastomotic leak than absolute values alone. J Clin Med 2025;14:2931. 10.3390/jcm14092931 PMC12072654 40363964

[10] de Wit A Daams F. Considerations in case of suspected anastomotic leakage in the lower GI tract. Best Pract Res Clin Gastroenterol 2024;70:101925. 10.1016/j.bpg.2024.101925 39053975

[11] Ochs V Taha-Mehlitz S Lavanchy JL. Development of a clinical prediction model for anastomotic leakage in colorectal surgery. JAMA Netw Open 2025;8:e2538267. 10.1001/jamanetworkopen.2025.38267 PMC12538359 41114976

